# Supplementary material for: Crystallographic observation of nonenzymatic RNA primer extension
Source: eLife. 2018 May 31;7:e36422. doi: 10.7554/eLife.36422 (PMC5980232; doi:10.7554/eLife.36422)
Supplement: Supplementary file 1. — Table S2: Structure refinement statistics. [file elife-36422-supp1.docx]

**Supporting Information**

**Crystallographic observation of nonenzymatic RNA primer extension**

Wen Zhang^1,2^, Travis Walton^1,2^, Li Li^1,2^, Jack W. Szostak^1,2,*,^

^1^Howard Hughes Medical Institute, Department of Molecular Biology and Center for Computational and Integrative Biology, Massachusetts General Hospital, 185 Cambridge Street, Boston, Massachusetts 02114, United States

^2^Department of Genetics, Harvard Medical School, 77 Avenue Louis Pasteur, Boston, Massachusetts 02115, United States

**Table S1. Data collection statistics.**

| **Structure** | RNA-dGMP | RNA-Gp-AI-pG | RNA-2-AIpG (5min) | RNA-2-AIpG (15min) |
| --- | --- | --- | --- | --- |
| **PDB code** | 6C8D | 6C8E | 6C8I | 6C8J |
| **Space group** | P3_1_21 | P3_1_21 | P3_1_21 | P3_1_21 |
| **Unit cell parameters (Å, ^o^)** | 49.54, 49.54, 81.34 90, 90, 120 | 49.02, 49.02, 81.32 90, 90, 120 | 43.84, 43.84, 85.22 90, 90, 120 | 49.09, 49.09, 81.10 90, 90, 120 |
| **Resolution range, Å (last shell)** | 50-1.92 (1.99-1.92) | 50-1.80 (1.86-1.80) | 50-1.77 (1.83-1.77) | 50-1.50 (1.55-1.50) |
| **Unique reflections** | 9264 (925) | 10695 (1051) | 9699 (965) | 16980 (1761) |
| **Completeness, %** | 99.9 (100) | 97.7 (100) | 99.1 (99.8) | 90.1 (95.7) |
| ***R*_merge_, %** | 14.2 (72.2) | 9.8 (71.3) | 12.7 (68) | 11.0 (55.3) |
| **<I/σ(I)>** | 12.6 (2.6) | 20.7 (2.95) | 14.2 (1.95) | 12.4 (3.8) |
| **Redundancy** | 7.9 (8.0) | 10.1 (9.9) | 7.6 (7.2) | 10.6 (9.1) |

| **Structure** | RNA-2-AIpG (30min) | RNA-2-AIpG (1h) | RNA-2-AIpG (1.5h) | RNA-2-AIpG (2h) |
| --- | --- | --- | --- | --- |
| **PDB code** | 6C8K | 6C8L | 6C8M | 6C8N |
| **Space group** | P3_1_21 | P3_1_21 | P3_1_21 | P3_1_21 |
| **Unit cell parameters (Å, ^o^)** | 48.10, 48.10, 82.20 90, 90, 120 | 47.95, 47.95, 82.39  90, 90, 120 | 47.87, 47.87, 82.73 90, 90, 120 | 46.98, 46.98, 83.10 90, 90, 120 |
| **Resolution range, Å**  **(last shell)** | 50-2.26 (2.34-2.26) | 50-2.25 (2.33-2.25) | 50-2.40 (2.49-2.40) | 50-1.90 (1.97-1.90) |
| **Unique reflections** | 4919 (245) | 5038 (255) | 4326 (428) | 8006 (402) |
| **Completeness, %** | 89.4 (46.2) | 90.6 (47.7) | 94.1 (96.4) | 91.6 (47.2) |
| ***R*_merge_, %** | 9.4 (43.1) | 6.8 (42.6) | 10.4 (73.2) | 7.9 (56.7) |
| **<I/σ(I)>** | 29.3 (2.4) | 34.7 (2.3) | 10.6 (1.2) | 24.5 (1.34) |
| **Redundancy** | 9.3 (7.7) | 9.5 (7.8) | 2.7 (2.6) | 9.0 (5.1) |

| **Structure** | RNA-2-AIpG (3h) | RNA-GMP (Sr^2+^) |
| --- | --- | --- |
| **PDB code** | 6C8O | 6CAB |
| **Space group** | P3_1_21 | P3_1_21 |
| **Unit cell parameters (Å, ^o^)** | 46.56, 46.56, 83.34 90, 90, 120 | 48.71, 48.71, 80.58 90, 90, 120 |
| **Resolution range, Å**  **(last shell)** | 50-1.85 (1.92-1.85) | 50-2.50 (2.59-2.50) |
| **Unique reflections** | 9369 (888) | 4047 (328) |
| **Completeness, %** | 99.7 (97.7) | 97.5 (82.0) |
| ***R*_merge_, %** | 10.1 (51.4) | 9.6 (44.0) |
| **<I/σ(I)>** | 21.9 (2.4) | 13.1 (1.96) |
| **Redundancy** | 8.6 (5.5) | 4.5 (3.1) |

**Table S2. Structure refinement statistics.**

| **Structure** | RNA-dGMP | RNA-Gp-AI-pG | RNA-2-AIpG (5min) | RNA-2-AIpG (15min) |
| --- | --- | --- | --- | --- |
| **Molecules per asymmetric unit** | 1 | 1 | 1 | 1 |
| **Resolution range, Å (last shell)** | 50-1.92 | 42.46-1.80 | 50-1.77 | 50-1.5 |
| ***R*_work_, %** | 21.9 | 26.7 | 23.0 | 21.1 |
| ***R*_free_, %** | 28.2 | 28.8 | 28.3 | 24.4 |
| **Number of reflections** | 8674 | 10123 | 9234 | 16084 |
| **Bond length R.M.S., Å** | 0.024 | 0.026 | 0.025 | 0.030 |
| **Bond angle R.M.S.** | 2.987 | 3.198 | 3.58 | 3.237 |
| **Average B-factors, Å^2^** | 34.07 | 53.53 | 31.6 | 28.72 |

| **Structure** | RNA-2-AIpG (30min) | RNA-2-AIpG (1h) | RNA-2-AIpG (1.5h) | RNA-2-AIpG (2h) |
| --- | --- | --- | --- | --- |
| **Molecules per asymmetric unit** | 1 | 1 | 1 | 1 |
| **Resolution range, Å (last shell)** | 50-2.26 | 50-2.25 | 50-2.40 | 50.00-1.90 |
| ***R*_work_, %** | 22.7 | 16.9 | 23.9 | 24.5 |
| ***R*_free_, %** | 30.2 | 25.3 | 27.2 | 30.6 |
| **Number of reflections** | 4521 | 4687 | 4006 | 7516 |
| **Bond length R.M.S., Å** | 0.020 | 0.025 | 0.021 | 0.025 |
| **Bond angle R.M.S.** | 2.624 | 2.984 | 2.773 | 2.660 |
| **Average B-factors, Å^2^** | 63.4 | 62.48 | 63.2 | 38.26 |

| **Structure** | RNA-2-AIpG (3h) | RNA-GMP (Sr^2+^) |
| --- | --- | --- |
| **Molecules per asymmetric unit** | 1 | 1 |
| **Resolution range, Å (last shell)** | 50.00-1.85 | 50.00-2.50 |
| ***R*_work_, %** | 23.1 | 21.5 |
| ***R*_free_, %** | 28.2 | 28.0 |
| **Number of reflections** | 8840 | 3729 |
| **Bond length R.M.S., Å** | 0.024 | 0.021 |
| **Bond angle R.M.S.** | 2.783 | 2.668 |
| **Average B-factors, Å^2^** | 36.81 | 73.72 |
